# A stilbene synthase allele from a Chinese wild grapevine confers resistance to powdery mildew by recruiting salicylic acid signalling for efficient defence

**DOI:** 10.1093/jxb/erw351

**Published:** 2016-10-11

**Authors:** Yuntong Jiao, Weirong Xu, Dong Duan, Yuejin Wang, Peter Nick

**Affiliations:** ^1^College of Horticulture, Northwest A & F University, Yangling 712100, Shaanxi, People’s Republic of China; ^2^Key Laboratory of Horticultural Plant Biology and Germplasm Innovation in Northwest China, Ministry of Agriculture, Yangling 712100, Shaanxi, People’s Republic of China; ^3^State Key Laboratory of Crop Stress Biology in Arid Areas, Northwest A & F University, Yangling, Shaanxi 712100, People’s Republic of China; ^4^Molecular Cell Biology, Botanical Institute 1, Karlsruhe Institute of Technology, Kaiserstr. 2, D-78133 Karlsruhe, Germany

**Keywords:** Basal immunity, defence, grapevine (*Vitis pseudoreticulata*), powdery mildew, promoter activity, SA, signalling, stilbene synthase.

## Abstract

Stilbenes are central phytoalexins in *Vitis*, and induction of the key enzyme stilbene synthase (STS) is pivotal for disease resistance. Here, we address the potential for breeding resistance using an *STS* allele isolated from Chinese wild grapevine *Vitis pseudoreticulata* (*VpSTS*) by comparison with its homologue from *Vitis vinifera* cv. ‘Carigane’ (*VvSTS*). Although the coding regions of both alleles are very similar (>99% identity on the amino acid level), the promoter regions are significantly different. By expression in Arabidopsis as a heterologous system, we show that the allele from the wild Chinese grapevine can confer accumulation of stilbenes and resistance against the powdery mildew *Golovinomyces cichoracearum*, whereas the allele from the *vinifera* cultivar cannot. To dissect the upstream signalling driving the activation of this promoter, we used a dual-luciferase reporter system in a grapevine cell culture. We show elevated responsiveness of the promoter from the wild grape to salicylic acid (SA) and to the pathogen-associated molecular pattern (PAMP) flg22, equal induction of both alleles by jasmonic acid (JA), and a lack of response to the cell death-inducing elicitor Harpin. This elevated SA response of the *VpSTS* promoter depends on calcium influx, oxidative burst by RboH, mitogen-activated protein kinase (MAPK) signalling, and JA synthesis. We integrate the data in the context of a model where the resistance of *V. pseudoreticulata* is linked to a more efficient recruitment of SA signalling for phytoalexin synthesis.

## Highlight

The resistance of the Chinese wild grapevine *Vitis pseudoreticulata* to powdery mildew is shown to differ from effector-triggered immunity by recruiting salicylic acid into phytoalexin synthesis based on a specific *stilbene synthase* promoter.

## Introduction

As one of the most ancient crops, grapevine (*Vitis vinifera* L.) is of economic and cultural significance. However, this crop is challenged by several diseases such as downy and powdery mildew, leading to a requirement for intensive plant protection. For instance, ~70% of European fungicide production is used for viticulture (Eurostat, 2007). This expensive application produces a negative ecological footprint, and is far from sustainable due to the rapid spread of fungicide resistance through the pathogen population. So far, resistance breeding has been the most successful strategy for sustainable viticulture and cost reduction (Eibach *et al.*, 2007). Resistance factors from non-*vinifera* species, such as the locus *Rpv3* (for Resistance to *Plasmopara viticola*) or the locus *Ren3* (for Resistance to *Erysiphe necator*) from North American wild grapes have been used successfully for introgression into *V. vinifera* cultivars that are used commercially (Welter *et al.*, 2007; Di Gaspero *et al.*, 2012). However, the success of these resistant varieties is progressively challenged by the occurrence of new pathogen strains that have already acquired strategies to circumvent the resistance mediated by these resistance loci (Peressotti *et al.*, 2010; Gómez-Zeledón *et al.*, 2013), stimulating the search for alternative mechanisms of resistance.

Plant innate immunity consists of two layers (Jones and Dangl, 2006; Boller and He, 2009) that have to be understood from the co-evolutionary interaction between host and pathogen. The more ancient pathogen-associated molecular pattern (PAMP)-triggered immunity (PTI) is activated by molecules termed PAMPs or microbe-associated molecular patterns (MAMPs), which are ubiquitous in pathogenic microorganisms and can be recognized by receptors at the plasma membrane. During co-evolution with their hosts, many pathogens have developed chemical signals, so-called effectors, to quell PTI. This will allow them to reinstate pathogenicity, but it will also initiate a second cycle of warfare, whereby the host cell can recognize the effectors by resistance proteins, predominantly nucleotide-binding-leucine-rich repeat (NB-LRR) receptors, and initiate a second layer of defence, termed effector-triggered immunity, or ETI (Takken and Tameling, 2009). This ETI often (but not necessarily) culminates in a hypersensitive response (HR) of the infected cell as the most efficient strategy to contain the intruder.

The canonical form of ETI is linked with specific strains of the pathogen that infect specific host genotypes based on a co-evolutionary history. In fact, both downy and powdery mildew have co-evolved with wild grapevine species in North America, and these grapevines have evolved resistance accompanied by HR. When these highly advanced biotrophic pathogens were unintentionally introduced into Europe in the second half of the 19th century, they encountered the completely naive host *V. vinifera* with devastating economic consequences. For downy mildew, the existence of ETI in *sensu stricto* is still under debate, but is supported by the recent discovery of host-specific pathogen strains (Gómez-Zeledón *et al.*, 2013; Rouxel *et al.*, 2013). In contrast, strain specificity for germplasm derived from wild North American grapes has been shown repeatedly for powdery mildew (Ramming *et al.*, 2012; Feechan *et al.*, 2015), supporting the existence of an ETI in *senso strictu*.

There is a current debate about the existence of ETI against powdery mildew in non-American grapes (Qiu *et al.*, 2015). This debate is mainly stimulated by claims of cell death-related defence resembling a HR. It should be kept in mind, however, that ETI has to meet three criteria: (i) it originates from a co-evolutionary history between host and pathogen; (ii) it is strain specific; and (iii) the cell death is not necrotic, but programmed. The first criterion is certainly not met, because powdery mildew arrived in China only in the 1950s (Wang, 1993; Wang *et al.*, 1995). The second criterion would require a study where different strains of powdery mildew are compared, and has, to the best of our knowledge, only been addressed and confirmed for the North American resistance loci (Ramming *et al.*, 2012; Feechan *et al.*, 2015). Even the third criterion, cell death of the programmed type, is controversial. For instance, the factor *Ren4* identified in the Chinese species *V. romanetii* confers a strain-independent resistance based on penetration barriers (Ramming *et al.*, 2010). However, in a recent review (Qiu *et al.*, 2015), unpublished data on *Ren4* are mentioned that are claimed to show a programmed cell death-based mechanism.

Also, VvPEN1 was proposed to cause penetration resistance against incompatible strains of powdery mildew (Feechan *et al.*, 2013*b*). Although only wild North American grapevines have co-evolutionary history with downy and powdery mildew, the use of non-American *Vitis* germplasm has great potential. For instance, the resistance loci, *Ren6* and *Ren7*, from the wild Chinese grapevine species *V. piasezkii* confer good immunity to powdery mildew. Whether this immunity is caused by programmed cell death as claimed by the authors of that study (Pap *et al.*, 2016) does not clearly verify the fact that such factors are highly valuable. Even if this, still unpublished, claim is substantiated, it would remain to be elucidated whether there is strain specificity as a central pre-condition for ETI. The discovery of a resistance locus, *Ren1*, in two local grapevine varieties ‘Kishmish Vatkana’ and ‘Kara Djandal’ from Usbekistan (Hoffmann *et al.*, 2008), that later turned out to be linked to a region rich in NB-LRR genes, has fostered speculation about the presence of ETI in this Non-American germplasm, leading to the suggestion of a potential and hitherto overlooked co-evolution with powdery mildew (Riaz *et al.*, 2013). The history of these varieties that derive from the Sultanina/Thompson Seedless lineage has been described in detail in Coleman *et al.* (2009). Most NB-LRR genes encode R-proteins, and have been proposed to derive from genes that had been recruited into a defence context (Ellis and Jones, 1998). In fact, functions of NB-LRR proteins in cytokinin signalling (Igari *et al.*, 2008) or phytochrome-dependent shade avoidance (Faigón-Soverna *et al.*, 2006) support a scenario where this evolutionarily ancient class of protein had undergone a functional shift from developmental signalling (possibly competitor sensing) to immunity (reviewed in Johnson *et al.*, 2003). Thus, while the presence of an NB-LRR gene in the *Ren1* locus triggering a cell death-related immunity provides an attractive possibility, alternative mechanisms should also be considered. For instance, the *Ren1* locus also harbours a cinnamyl alcohol dehydrogenase (Coleman *et al.*, 2009), a member of the phenylpropanoid pathway that has been shown to be an important resistance factor against powdery mildews, because it is crucial for papilla formation (Bhuiyan *et al.*, 2009). Thus, whether ETI for powdery mildew exists outside of the Non-American grapes remains to be elucidated. However, it should be kept in mind that ETI is not the only path to success, and a co-evolutionary history is not a *conditio sine qua non* for resistance.

Although the receptors triggering PTI and ETI are thought to differ, the molecular events underlying signalling are partially shared: these include calcium influx, activation of the apoplastic oxidative burst, MAPK (mitogen-activated protein kinase) cascades, and transcriptional activation (Schwessinger and Zipfel, 2008; Nürnberger and Kemmerling, 2009; Tsuda and Katagiri, 2010). However, PTI usually does not lead to programmed cell death, whereas ETI in most (but not in all) cases does (Thomma *et al.*, 2011). Comparative studies in grapevine cells have revealed that the differential output of defence is associated with differences in the relative timing of the early signalling events (Chang and Nick, 2012; Chang *et al.*, 2016). The phytohormones jasmonic acid (JA) and salicylic acid (SA) have been documented to play central roles in this context. The JA pathway is mainly induced by and involved in regulating resistance against herbivores and necrotrophic pathogens (i.e. a type of defence where programmed cell death is not observed). Also in grapevine cell cultures, accumulation of jasmonates was only observed in PTI triggered by the PAMP flg22, but not in cell death-related immunity triggered by the elicitor Harpin (Chang *et al.*, 2016). In contrast, the SA pathway is primarily activated by and involved in mediating resistance against biotrophic pathogens (i.e. under conditions where hypersensitive cell death occurs; Glazebrook, 2005). SA is also required for systemic acquired resistance (SAR) (Durrant and Dong, 2004).

Stilbenes, as the central grapevine phytoalexins, confer resistance to a broad spectrum of pathogens (Adrian *et al.*, 1997; Schnee *et al.*, 2008). In particular, the non-glycosylated resveratrol has attracted increasing attention for its medical benefits (Roupe *et al.*, 2006). The first product of the committed stilbene branch of the phenylpropanoid pathway is synthesized from coumaroyl-CoA and malonyl-CoA by the enzyme stilbene synthase (STS). This key enzyme belongs to the type-III group of the polyketide synthase enzyme superfamily, which has strongly expanded in grapevine with 48 members, at least 32 of which are potentially functional (Parage *et al.*, 2012). The large number of *STS* genes with potential functions already indicates the importance of stilbenes for defence in grapevine. In fact, an extensive screen in *V. sylvestris*, the wild ancestor of the cultivated grapevine *V. vinifera*, demonstrated a correlation between the inducibility of resveratrol accumulation and performance against infection with downy mildew (Duan *et al.*, 2015). In addition, the relationships between susceptible and resistant grapevine varieties according to stilbene concentrations in response to powdery mildew had been described by Schnee *et al.* (2008).

However, the induction of *STS* is not confined to basal immunity, but is also observed in the context of cell death-related defence in grapevine (Chang and Nick, 2012). *STS* alleles with elevated responsiveness of their promoters might therefore be an interesting target for future resistance breeding. Non-American wild species of *Vitis* have not co-evolved with downy or powdery mildew and thus are unlikely to launch ETI directed against these pathogens. Nevertheless, the above-mentioned factors *Ren6* and *Ren7* (Pap *et al.*, 2016), *Ren4* (Ramming *et al.*, 2010; Qiu *et al.*, 2015), and *Ren1* (Hoffmann *et al.*, 2008; Coleman *et al.*, 2009) provide examples for such factors conferring resistance to powdery mildew.

Already more than two decades ago, 18 *Vitis* species native to China were probed for resistance to powdery mildew (Wang *et al.*, 1995). This approach had uncovered a genotype in the Chinese wild grape *V. pseudoreticulata* which was resistant to powdery mildew (Wang *et al.*, 1995). From this genotype, a specific *STS* allele was isolated that differed upon heterologous expression in tobacco as monitored by a β-glucuronidase (GUS) reporter. The *STS* promoter from *V. pseudoreticulata* was not induced by methyl jasmonate (MeJA), and was not responsive to the necrotrophic pathogen *Alternaria alternata* (Xu *et al.*, 2011). However, the same promoter was activated after inoculation with the biotrophic pathogen *Erysiphe necator* (the causative agent of grapevine powdery mildew) or by treatment with SA (Xu *et al.*, 2010). In contrast, the *STS* promoters from the *vinifera* cultivars ‘Carigane’ and ‘Thompson Seedless’ showed the inverse pattern of regulation.

When expression of different *STS* alleles can be differentially activated either by necrotrophic pathogens/JA or by biotrophic pathogens/SA, this leads to the question of whether *STS* promoters can differentially recruit JA and SA signalling, whether activation of *STS* is sufficient to ward off biotrophic pathogens, at what point the two signal chains converge, and which signalling events differ between them. We addressed these questions using two strategies. First, we introduced the *STS* promoters of *V. pseudoreticulata* and the reference promoter from the *V. vinifera* cultivar ‘Carigane’ driving an *STS* coding sequence into Arabidopsis as a heterologous system otherwise not capable of stilbene synthesis. In the second approach, we analysed the two promoters in a homologous promoter–reporter system (a *V. vinifera* cell culture) to dissect the upstream signals conferring the response to SA. We provide evidence for a model where STS can be activated by two pathways that both employ the signalling chain driving basal immunity. One pathway passes through activation of *MYB14* and depends on jasmonate, and the second pathway acts independently of *MYB14* and integrates SA into basal immunity. This second pathway is more efficiently recruited by the *STS* allele from the wild Chinese grapevine *V. pseudoreticulata*.

## Materials and methods

### Plant materials

The powdery mildew-resistant Chinese wild *V. pseudoreticulata* accession Baihe-35-1 (Wang *et al.*, 1995) and the powdery mildew-susceptible *V. vinifera* cv. ‘Carigane’ were cultivated in the Grape Repository of Northwest A&F University, Yangling, Shaanxi, China. Arabidopsis ecotype Columbia were grown on Murashige and Skoog (MS) medium agar plates at 22 °C for 10–14 d, then transferred into a mix of peat moss, perlite, and vermiculite (3:1:1, by vol.), and cultivated under a 16h light/8h dark regime at 22 °C with 75% humidity.

### Isolation of *STS* promoter fragments and plasmid constructs

Genomic DNA from the leaves of *V. pseudoreticulata* and *V. vinifera* cv. ‘Carigane’ was extracted using the CTAB (cetyltrimethylammonium bromide) protocol (Lodhi *et al.*, 1994) and then used as template. Two fragments encoding the *STS* coding region and the respective native upstream promoter were amplified by PCR using the primers (Supplementary Table S1 at *JXB* online) designed according to Xu *et al.* (2011). Their chromosomal location was confirmed by a BLAST search in the Genoscope Genome Browser (http://www.genoscope.cns.fr/blat-server/cgi-bin/vitis/webBlat). Both genomic fragments were inserted into the pART-CAM-S vector (Xu *et al.*, 2014) after restriction by *Xho*I and *Sac*I. From the amplified genomic sequences, one sense primer and two antisense primers (Supplementary Table S1) were designed to isolate the *STS* promoters. The verified promoter sequences were analysed for the presence of putative *cis*-elements using the PLACE (plant *cis*-acting regulatory DNA elements) algorithms. Subsequently, both amplified promoter fragments were cloned into the *Sac*I/*Nco*I site of pCAMBIA1301 (http://www.cambia.org/daisy/cambia/585.html). Each of these constructs was individually introduced into *Agrobacterium tumefaciens* strain GV3101 by electroporation (Mersereau *et al.*, 1990). To conduct the transient luciferase assay, the *STS* promoter regions were amplified with Q5 High-Fidelity DNA Polymerase (NEB) using the specific primers (Supplementary Table S1) to generate attB-PCR products. After Gateway^®^ BP and LR recombination, the promoter regions of *VpSTS* and *VvSTS* were transferred into the pLuc luciferase vector (Horstmann *et al.*, 2004) and confirmed by DNA sequencing.

### Pathogen inoculation and microscopic analysis of colonization

*Golovinomyces cichoracearum* strain UCSC1 (Wen *et al.*, 2011) was maintained on living Arabidopsis mutant *pad4* plants to generate fresh inocula. Inoculation was carried out as previously described (Xiao *et al.*, 1997). Fungal structures in inoculated leaves were stained blue by trypan blue and observed by bright-field microscopy (Xiao *et al.*, 2003).

### Arabidopsis transformation and GUS assays

Arabidopsis transformation was carried out using the floral dipping method (Clough and Bent, 1998). The T_3_ generation of transgenic Arabidopsis lines were inoculated either with aseptic water or with the pathogen and then used for histochemical and quantitative assays of GUS activity as described by Jefferson *et al*. (1987); experiments were carried out in triplicate using three independent lines.

### Quantification of gene expression by qRT-PCR

Total RNA was extracted from leaf samples of transgenic Arabidopsis using the EZNA^®^ Total RNA kit II (Omega Bio-tech), and then transcribed into cDNA with Prime Script Reverse Transcriptase (TaKaRa) according to the manufacturer’sְ instructions. Real-time quantitative PCR (qRT-PCR) was conducted by a two-step protocol as described in Xu *et al.* (2011), using *AtGAPDH* (AT1G13440) as the internal standard. Primers used in qPCR experiments are listed in Supplementary Table S1; experiments were repeated three times.

### Biolistic transformation and treatment

The grapevine cell suspension culture *V. vinifera* cv. ‘Pinot Noir’ (Seibicke, 2002) cultivated in MS medium was collected at day 4 after subcultivation (just at the end of the cycling phase) and transiently transformed by particle bombardment as described by Maisch *et al.* (2007). After expression for 48h, the transgenic suspension cells were subjected to the various treatments before assaying the luciferase activity. All transfection experiments were carried out in triplicate and each set of promoter experiments was repeated at least twice.

For the induction treatments, the bacterial peptide flg22, the bacterial elicitor Harpin, SA, and MeJA were prepared as described previously (Duan *et al.*, 2016). The UV-C induction was performed as described by Duan *et al.* (2015). For inhibition treatments, the transgenic suspension cells were pre-treated with the respective inhibitors for 30min before SA was administered. 2-(2-Amino-3-methoxyphenyl)-4H-1-benzopyran-4-one (PD98059), diphenylene iodonium chloride (DPI), gadolinium chloride (GdCl_3_), and phenidone were prepared as described by Duan *et al.* (2016). All experiments were accompanied by controls, where each inhibitor was added without SA to assess the impact of inhibitors on cells. All treatments were accompanied by solvent controls, with the maximal concentration of the solvent not exceeding 0.1%.

### Dual-luciferase assay

After the transgenic suspension cells had been treated for 6h at 22 °C in the dark, the harvested cells were collected to measure luciferase activities using the dual-luciferase reporter assay system as described previously (Czemmel *et al.*, 2009; Höll *et al.*, 2013). The relative luciferase activity was calculated as mean values of firefly and *Renilla* luciferase ratios after subtraction of the cell background (cells that had not been bombarded).

## Results

### Isolation and analysis of two *STS* alleles and their promoters

**Fig. 1.**
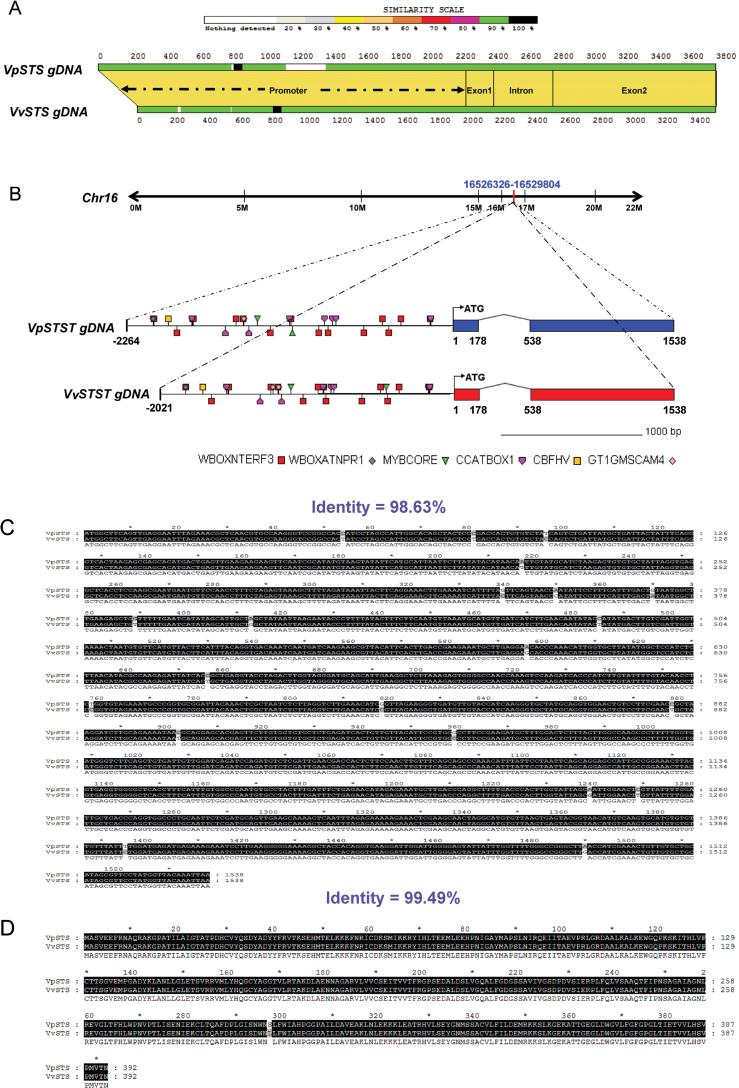
Similarity pattern, chromosome location, and *cis*-element analysis for two alleles of a stilbene synthase from the Chinese wild *Vitis pseudoreticulata* accession Baihe-35-1 (*VpSTS*) in comparison with the *Vitis vinifera* cv. ‘Carigane’ (*VvSTS*). (A) Similarity patterns of the two *STS* alleles showing high conservation in the transcribed regions, but specific differences in the promoter region. (B) Chromosomal location and predicted *cis*-elements of the two *STS* alleles based on the reference genome from *Vitis vinifera* cv. ‘Pinot Noir’ clone P40024 (Jaillon *et al.*, 2007). The triangles represent MYBCORE; the pentagons represent CCAATBOX1; the squares represent WBOXNTERF3 except the second one which is CBFHV; and the rhombuses represent GT1GMSCAM4 except the first one which is WBOXATNPR1. (C and D) Alignment of the nucleotide and amino acid sequences for the two *STS* alleles showing a high degree of conservation. (This figure is available in colour at *JXB* online.)

To verify the different patterns of expression and hormonal regulation observed for the STSs from the Chinese wild *V. pseudoreticulata* accession Baihe-35-1 versus *V. vinifera* cv. ‘Carigane’, genomic fragments containing the *STS* coding region and its native promoter were amplified by PCR using genomic DNA isolated from the two genotypes. The PCR-ampliﬁed fragment of *V. pseudoreticulata* (*VpSTS*) was 3802bp in length, of which the promoter accounted for 2264bp, while the fragment amplified from *V. vinifera* cv. ‘Carigane’ (*VvSTS*) was significantly shorter (3559bp), due to a shorter (2021bp) promoter. Using the Genoscope Genome Browser, both amplified fragments could be assigned to the same locus in chromosome 16 (Fig. 1B). The alignment revealed a highly conserved coding region with 98.63% identity at the nucleotide level and 99.49% identity at the amino acid level (Fig. 1A, C, D). In contrast, the promoter sequences, despite some degree of congruence, displayed specific differences. In particular, the promoter of the wild allele (*VpSTS*) harboured two stretches (of 13bp and 244bp, respectively) that were absent in the *VvSTS* allele, whereas the *VpSTS* allele lacked a stretch of 18bp found in *VvSTS*. Comparison of the two promoters with respect to predicted *cis*-regulatory elements revealed that both promoter alleles shared somef putative stress-responsive elements: WBOXATNPR1 was reported to confer a response to SA (Yu *et al.*, 2001); WBOXNTERF3 has been shown to drive response to wounding (Nishiuchi *et al.*, 2004); CCAATBOX1 is linked to responsiveness to heat (Rieping *et al.*, 1992); CBFHV has been shown to be the binding site for CBF1 during the response to dehydration (Xue *et al*., 2002); MYBCORE was found to be involved in the regulation of flavonoid biosynthesis (Solano *et al.*, 1995); and GT1GMSCAM4 has been linked to the response to salt and biotic stress (Park *et al.*, 2004).

### The two *STS* promoter alleles respond differentially to powdery mildew

**Fig. 2.**
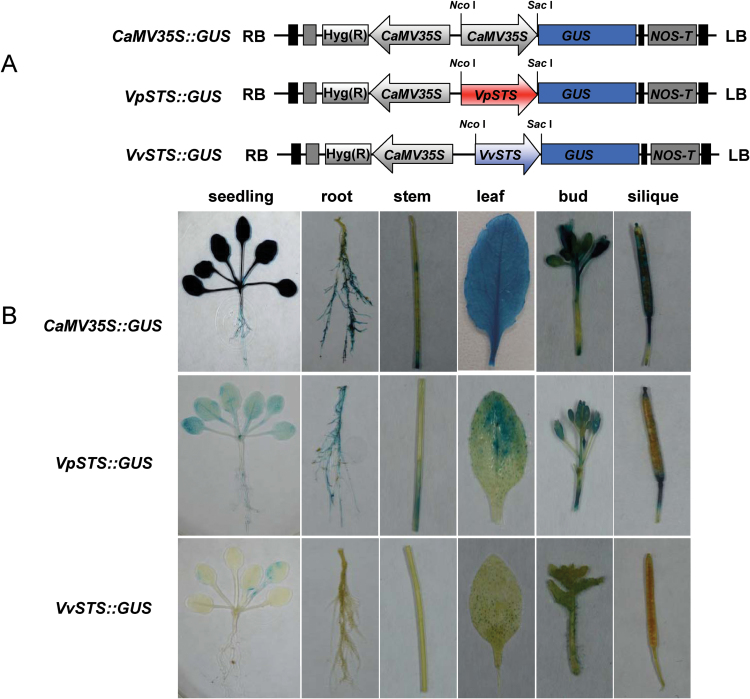
Basal promoter activities for the two stilbene synthase alleles (*VpSTS* versus *VvSTS*) upon heterologous expression in Arabidopsis driving a GUS reporter. (A) Schematic representation of the three promoter GUS fusion constructs. *CaMV-35S::GUS* was used as positive control; the putative *STS* promoter regions of two grapevine genotypes were inserted into the *Xho*I and *Sac*I sites of pCAMBIA1301 to generate *VpSTS::GUS* and *VvSTS::GUS*, respectively. GUS, β-glucuronidase; Nos-T, Nos terminator; RB, right border; LB, left border. (B) GUS staining of different tissues from the T_3_ transgenic Arabidopsis, including seedling stage, root, stem, leaf, bud, and silique to test basal GUS activity in the absence of pathogen inoculation. Experiments were performed in three biological replicates, and representative images from the staining of at least three independent lines are shown. (This figure is available in colour at *JXB* online.)

**Fig. 3.**
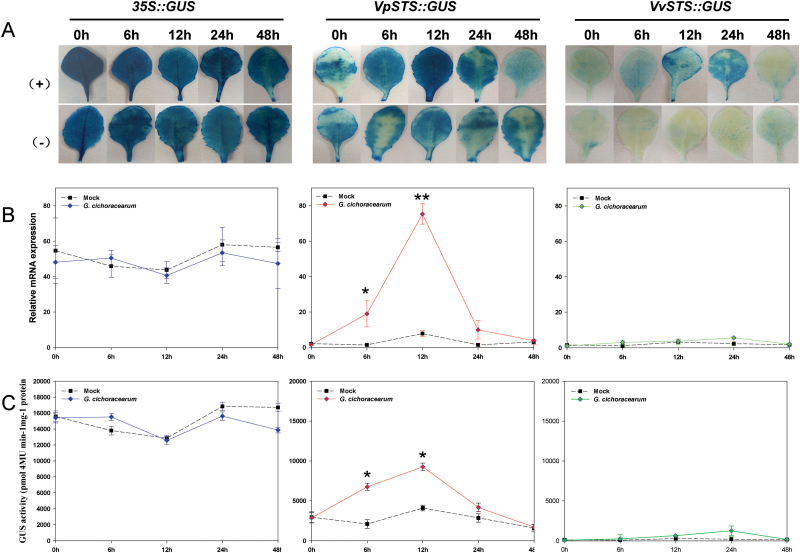
Time course for the accumulation of the GUS reporter upon heterologous expression of *CaMV-35S::GUS*, *VpSTS::GUS*, and *VvSTS::GUS* in transgenic Arabidopsis leaf tissues in response to inoculation with *Golovinomyces cichoracearum* strain UCSC1 for the two *STS* promoter alleles. (A) Histochemical detection of GUS activity in response to pathogen inoculation (+) compared with the mock control (–). (B) qRT-PCR of GUS transcripts. (C) Measurement of GUS enzymatic activity. (+), pathogen-inoculated leaves; (–), mock-inoculated leaves. The *GUS* transcripts in (B) were normalized to *GAPDH* as an internal reference. Results represent mean values and SEs from three independent transgenic lines. * and ** indicate statistical signiﬁcance of the difference from mock controls by a one-sided paired *t*-test with confidence levels of *P*<0.05 or *P*<0.01, respectively. (This figure is available in colour at *JXB* online.)

To test whether the structural differences between the *VpSTS* and *VvSTS* promoters are of functional relevance, the 2264bp (*VpSTS*) and the 2021bp (*VvSTS*) promoter fragments were inserted into *Sac*I and *Nco*I sites of pCAMBIA1301, replacing the *Cauliflower mosaic virus* (*CaMV*) *35S* promoter region and placed upstream of a GUS reporter, then transformed in a stable manner into Arabidopsis as the heterologous system using the floral dipping method (Clough and Bent, 1998). After selection by 25mg l^–1^ hygromycin, and verification of the transgene by genomic PCR, the basal expression was assessed (Fig. 2). In the next step, the response of the two promoters to a compatible strain of powdery mildew UCSC1 (*G. cichoracearum*) was analysed (Fig. 3). A *CaMV-35S::GUS* construct, used as a positive control (Fig. 2A), showed, as expected, a strong expression in all tested tissues including seedling, root, stem, leaf, bud, and silique. In contrast, the two *STS* promoters were active at a much weaker level, with *VpSTS* being active in all tissues, whereas the activity of the *VvSTS* promoter could only be measured in leaves and even here its activity was considerably lower than that of the *VpSTS* promoter (Fig. 2B).

To assess whether the *VpSTS* and *VvSTS* promoters can be induced by powdery mildew, GUS expression was followed by X-Gluc staining (Fig. 3A), qRT-PCR analysis of *GUS* transcripts (Fig. 3B), and quantification of GUS enzymatic activity (Fig. 3C) in leaves after inoculation with the compatible powdery mildew *G. cichoracearum* strain UCSC1 compared with a mock inoculation with aseptic water. The *CaMV-35S* promoter used as positive control yielded a constitutively strong GUS expression and did not reveal any significant difference between pathogen inoculation and water treatment. In contrast, the *VpSTS* promoter was significantly induced in response to *G. cichoracearum* (Fig. 3A). Quantification of *GUS* transcripts (Fig. 3B) and enzymatic activity (Fig. 3C) revealed a strong but transient induction detectable from 6 hours post-inoculation (hpi), and reaching its peak at 12 hpi, followed by a sharp decline approaching the low expression seen in the mock control. The maximal activity reached at 12 hpi was comparable with that achieved by the constitutive *CaMV-35S* promoter. In the mock control, there was a slight increase as well, but this was not statistically significant. In contrast to *VpSTS*, the *VvSTS* promoter produced only a slight induction. Again, this induction remained below the threshold for significance. These findings show that the *VpSTS* promoter not only confers a higher basal expression, but also is sufficient to produce inducibility by the compatible powdery mildew *G. cichoracearum* in the heterologous Arabidopsis system.

To test whether these differences in the promoter responses to infection by the biotrophic pathogen correlate with a differential response to SA and MeJA, respectively, we followed the accumulation of *GUS* transcripts after treatment with 1mM of either exogenous SA or exogenous MeJA. We observed that the *VpSTS* promoter was strongly and rapidly induced by SA: already at the first time point (which, due to the time required for excision, was ~2min after application of SA), the transcript was found to be slightly elevated; after 4h an almost 5-fold induction had been reached; and this high level of expression persisted over the entire 12h of this experiment. In contrast, the *VvSTS* promoter was induced to a much lower extent and more slowly: even after 8h the induction was only ~2-fold. For MeJA, both promoters responded more slowly compared with SA: here, the *VpSTS* promoter was induced weakly, but significantly, after 8h, and the *VvSTS* promoter after 12h (Supplementary Fig. S1).

### The full-length *VpSTS* allele is sufficient to confer resistance to powdery mildew in a heterologous system

**Fig. 4.**
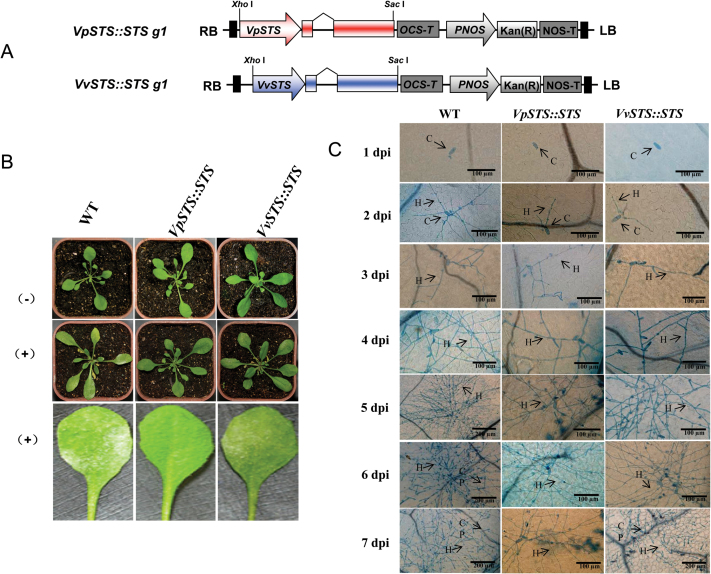
Time course of fungal colonization by *G. cichoracearum* in Arabidopsis expressing the two full-length alleles of *Vitis STS* under control of their native promoters. (A) Schematic representation of constructs used for stable transformation of *Arabidopsis thaliana.* (B) Expression of macroscopic disease symptoms in T_3_ transgenic Arabidopsis compared with the wild-type Col-0 after inoculation with *G. cichoracearum* at 7 dpi. (C) Time course of leaf colonization by *G. cichoracearum* followed by histology based on staining with trypan blue; the scale bars refer to each image. C, conidia; H, hyphae; CP, conidiophores. (This figure is available in colour at *JXB* online.)

**Fig. 5.**
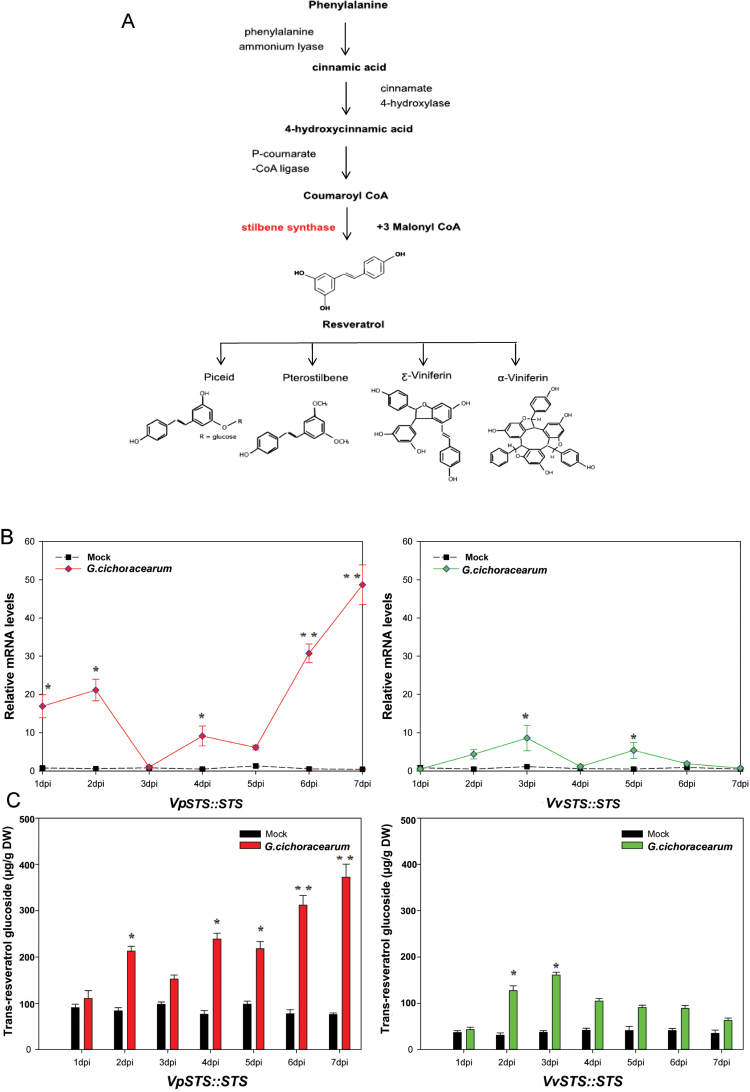
Time course for the expression of the *STS* reporter upon heterologous expression of *VpSTS::STS* compared with *VvSTS::STS* in response to inoculation with *G. cichoracearum*. (A) Chemical structures and biosynthetic pathway of stilbenes. Both mock-inoculated and pathogen-inoculated leaves were sampled at the indicated time points for the quantification of *STS* transcripts by qRT-PCR (B), and the resveratrol glucoside α-piceid (C). The housekeeping gene *AtGAPDH* (AT1G13440) was used as the internal standard for the transcript measurements (B). Values represent means and SEs of three biological replicates. * and ** indicate statistical signiﬁcance of the difference from mock controls by a one-sided paired *t*-test with confidence levels of *P*<0.05 or *P*<0.01, respectively. (This figure is available in colour at *JXB* online.)

To test whether the full-length *VpSTS* allele is sufficient to confer pathogen resistance, the two *STS* alleles (consisting of the full-length coding sequence under control of the respective native promoter) derived from *V. pseudoreticulata* and *V. vinifera* were inserted into the pART-CAM-S vector (Xu *et al.*, 2014) after restriction by *Xho*I and *Sac*I, and then transformed into Arabidopsis as the heterologous system (Fig. 4). Transformants verified by resistance to the hygromycin marker and genotyping by PCR were propagated, and the T_3_ generation was used to follow colonization by *G. cichoracearum* strain UCSC1 (Fig. 4) in parallel with the response of the *STS* transgene (Fig. 5) over 1 week. Whereas leaves of the wild type (WT) were covered with abundant sporangiophores at 7 days post-inoculation (dpi), lines expressing the complete alleles of *Vitis STS* exhibited lower sporulation densities, which was most pronounced for the *VpSTS* allele (Fig. 4B).

When colonization was followed over time by histology, fungal spores were found to have attached to the leaf surface successfully at 1 dpi in all three genotypes. Likewise, germination and appressoria formation (from 2 dpi) seemed to proceed normally. However, the progress of colonization was impaired from 4 dpi in the lines expressing the allele from *V. pseudoreticulata*, and from 5 dpi in the lines expressing the allele from *V. vinifera*. In contrast, *G. cichoracearum* continued colonization in the WT Col-0 and, from 5 dpi, the mycelium had almost covered the whole infected surface. The inhibition of colonization was preceded by accumulation of *STS* transcripts and formation of the stilbene glycoside α-piceid (Fig. 5B, C), particularly in the allele driven by the *VpSTS* promoter. In summary, the phenotype of the transgenic Arabidopsis lines showed that expression of full-length alleles of *STS* was correlated with arrested colonization of the compatible powdery mildew *G. cichoracearum*, and that this arrest was more pronounced for the allele derived from the wild Chinese species *V. pseudoreticulata*.

### The *VpSTS* promoter is more efficiently activated by basal immunity

**Fig. 6.**
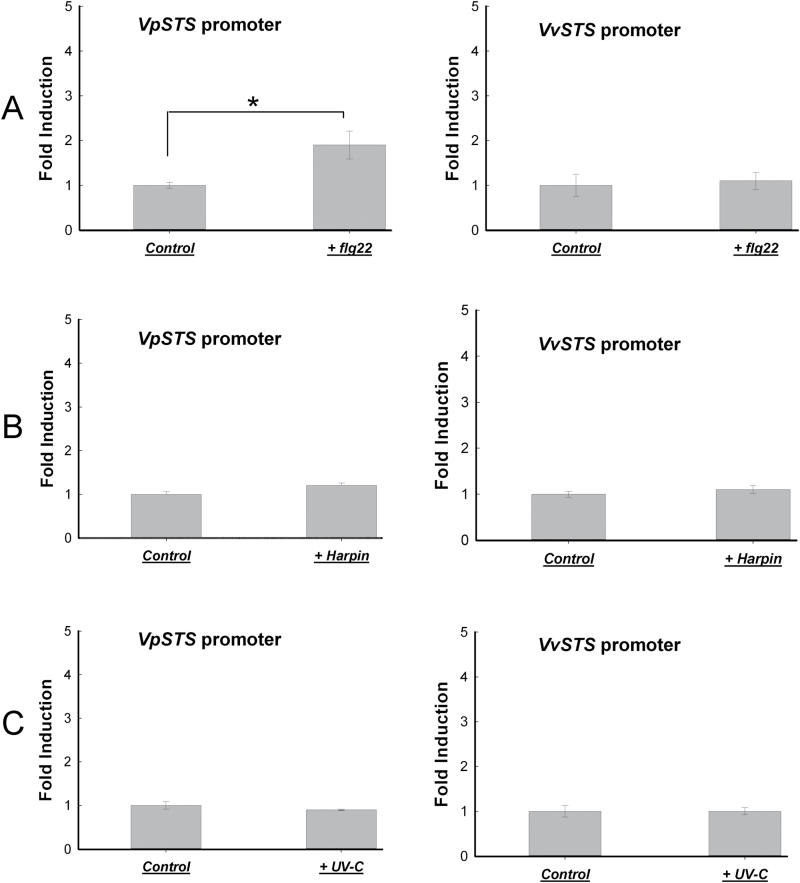
Activation of *VpSTS* and *VvSTS* promoters by flg22 in a grapevine suspension cell line derived from the *vinifera* cultivar ‘Pinot Noir’. Induction of promoter activity was measured either 6h after addition of 10 μg l^–1^ flg22 (A) or of 9 μg ml^–1^ Harpin (B), or after irradiation with UV-C for 2min (C). Data represent mean values and SEs from three biological replicates. * indicates statistical signiﬁcance of the difference from controls by a one-sided paired *t*-test with a confidence level of *P*<0.05.

The *STS* allele from *V. pseudoreticulata* had been found to confer pathogen resistance in the heterologous Arabidopsis system (Fig. 4), namely in a situation where no co-evolutionary history between pathogen and gene activation can definitely be expected. Likewise, a co-evolutionary context cannot account for the resistance of this wild Chinese *Vitis* species to powdery mildew of grapevine (*Erysiphe necator*), since this pathogen is native to North America and came to China only a few decades ago (Wang, 1993; Wang *et al.*, 1995). These considerations stimulated the question of whether the *STS* allele of *V. pseudoreticulata* is activated in the context of basal immunity (PTI), or whether it is linked rather to a cell death-related, ETI-like immunity. To dissect the signalling upstream of *STS*, we employed a dual-luciferase promoter reporter system in a *vinifera* cell culture (cv. ‘Pinot Noir’) as the homologous expression system (Höll *et al.*, 2013) using biolistic transformation. We used this genotype because ‘Pinot Noir’ under normal conditions cannot efficiently deploy a programmed cell death response that would overlay the effect of the introduced transgene. The transgenic suspension cells were treated either with 10 µg l^–1^ flg22, a potent inducer of basal immunity, or with 9 µg ml^–1^ of the bacterial elicitor Harpin, triggering a cell death-related ETI-like response in these cells (Chang and Nick, 2012). Alternatively, a short pulse (2min) of UV-C was used as an abiotic inducer of stilbene metabolism (Duan *et al.*, 2015). After induction by these three treatments, the cells were allowed to express the luciferase reporter for 6h in the dark before measuring the activity. Using this system, we observed that the *VpSTS* promoter was significantly (*P*<0.05) activated by the bacterial PAMP flg22 by ~1.9-fold, while this activation was not found for the *VvSTS* promoter. In contrast to flg22, neither the elicitor Harpin nor a pulse of UV-C (a powerful abiotic activator of stilbene accumulation in *Vitis*) was able to stimulate luciferase activity over the values observed in the controls (Fig. 6B, C).

### The *VpSTS* and *VvSTS* promoters are differentially induced by SA

**Fig. 7.**
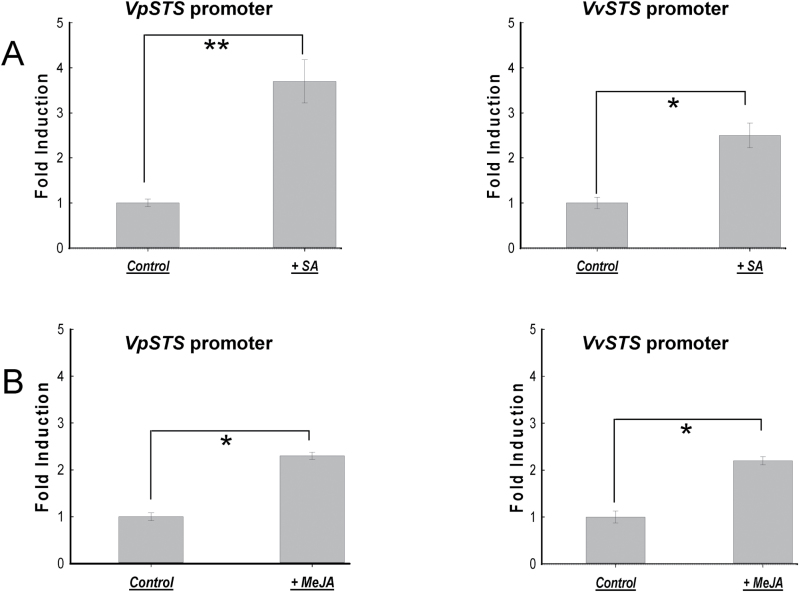
Activation of *VpSTS* and *VvSTS* promoters by 50 µM SA (A) and MeJA (B), respectively. Induction of promoter activity was measured 6h after addition of either 50 µM SA or MeJA. Data represent mean values and SEs from three biological replicates. * and ** indicate statistical signiﬁcance of the difference assessed by a one-sided paired *t*-test with confidence levels of *P*<0.05, or *P*<0.01, respectively.

As pointed out in the Introduction, defence against biotrophic pathogens has been associated with SA, whereas jasmonates have been shown to act in the defence against necrotrophic pathogens. We therefore tested the response of the two *STS* promoters to 50 µM of either SA or MeJA for 6h using the luciferase assay described above (Fig. 7). We found that SA activated the *VpSTS* promoter by 3.7-fold (*P*<0.01) compared with a solvent control (Fig. 7A), whereas the induction of the *VvSTS* promoter was significantly weaker (2.5-fold). In contrast, both promoters responded in a similar way to MeJA treatment (Fig. 7B). Here, the induction of the *VpSTS* promoter was only 2.3-fold, and that of the *VvSTS* promoter was 2.2-fold. Thus, the *VpSTS* promoter was found to be specifically responsive to SA, whereas both promoter alleles were not different with respect to their responses to MeJA.

### Induction of *VpSTS* and *VvSTS* promoters by SA depends on calcium influx and NADPH oxidase activity

**Fig. 8.**
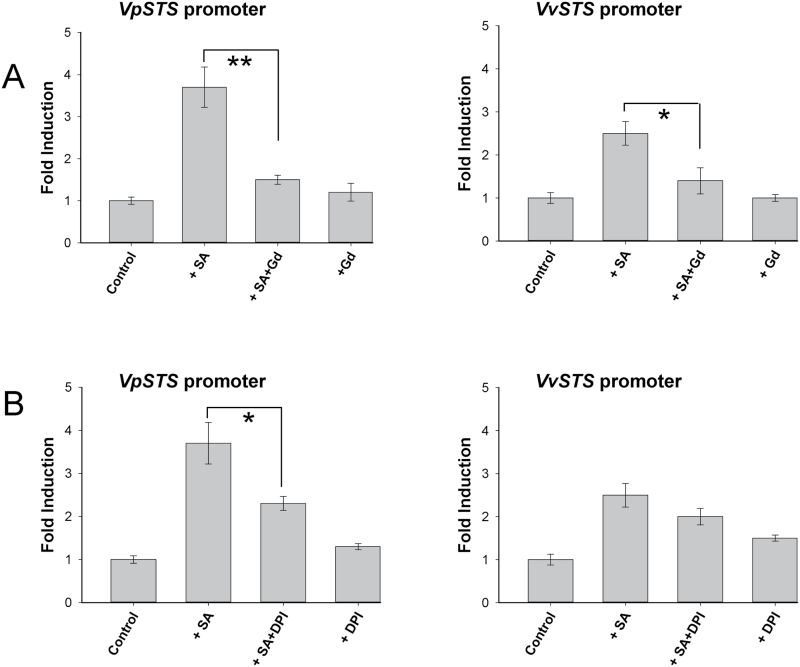
Regulation of *STS* promoters by salicylic acid (SA) depends on calcium influx and NADPH oxidase activity. Activation of *VpSTS* and *VvSTS* promoters by 50 µM SA was measured after pre-treatment for 30min with (A) 20 µM of the calcium influx blocker gadolinium chloride (Gd) or (B) 10 µM of the NADPH oxidase inhibitor diphenylene iodonium (DPI). The same concentrations of the two inhibitors without subsequent addition of SA were used as negative controls. Data represent mean values and SEs from three biological replicates. * and ** indicate statistical signiﬁcance of the difference assessed by a one-sided paired *t*-test with confidence levels of *P*<0.05 or *P*<0.01, respectively.

Previous work on grapevine suspension cells had shown that the transcripts of *STS* were induced by the elicitor Harpin dependent on a calcium influx channel and an apoplastic oxidative burst triggered by an NADPH oxidase (Chang *et al.*, 2011). We therefore tested the effect of GdCl_3_ (an inhibitor of calcium influx) as well as the NADPH oxidase inhbitor DPI on the activation of *STS* promoters by SA (Fig. 8). Whereas 20 µM GdCl_3_ by itself did not cause any modulation of promoter activity, this pre-treatment almost completely abolished the activation of the two *STS* promoters by SA (Fig. 8A). When the transformed cells were pre-treated with GdCl_3_ for 30min before the SA treatment, the induction of the *VpSTS* promoter was decreased from 3.7-fold to 1.5-fold, and the activity of the *VvSTS* promoter was decreased from 2.5-fold to 1.4-fold. Inhibition of NADPH oxidase by DPI had a similar but milder effect on SA inducibility of *VpSTS* promoter activity (Fig. 8B). Here, pre-treatment of the transgenic cells with DPI for 30min decreased the SA induction of *VpSTS* promoter activity from 3.7-fold to 2.3-fold, and from 2.5-fold to 2.0-fold for the *VvSTS* promoter, but the decrease remained below the threshold for significance. Again, the inhibitor alone did not produce any significant modulation. These results show that calcium influx is necessary for the induction of *VpSTS* and *VvSTS* promoters by SA, whereas apoplastic reactive oxidative species (ROS) generated by the NADPH oxidase act as positive modulators of the SA-dependent signalling significantly activating the *VpSTS* promoter. This positive modulation is different for the two promoter alleles, since the *VpSTS* promoter responded to SA more strongly than the *VvSTS* promoter, but both alleles show the same residual activation observed under elimination of NADPH oxidase.

To understand whether the signal generated by apoplastic ROS amplifies with SA-dependent activation of *STS* promoter activity at an early or a late state of signalling, a time course experiment was conducted where DPI was administered at different time points with respect to the inductive SA treatment (Supplementary Fig. S2). We observed that if the transgenic suspension cells were pre-treated with DPI for 30min before the SA treatment, or treated with SA and DPI at the same time, the induction of the *VpSTS* promoter by SA was obviously suppressed. The *VvSTS* promoter activity decreased as well, but, again, this decrease remained below the significance threshold. However, when the transgenic cells were treated with DPI after SA had been acting for 30min, the activation of both promoters was also decreased, and when administered 1h after SA treatment, DPI had little effect on the activation of *STS* promoters by SA. This indicates that ROS are effective up to 30min after application of SA, showing that they are not part of an early induction step but modulate a downstream event.

### Induction of *VpSTS* and *VvSTS* promoters by SA is modulated by the MAPK pathway and jasmonate synthesis

**Fig. 9.**
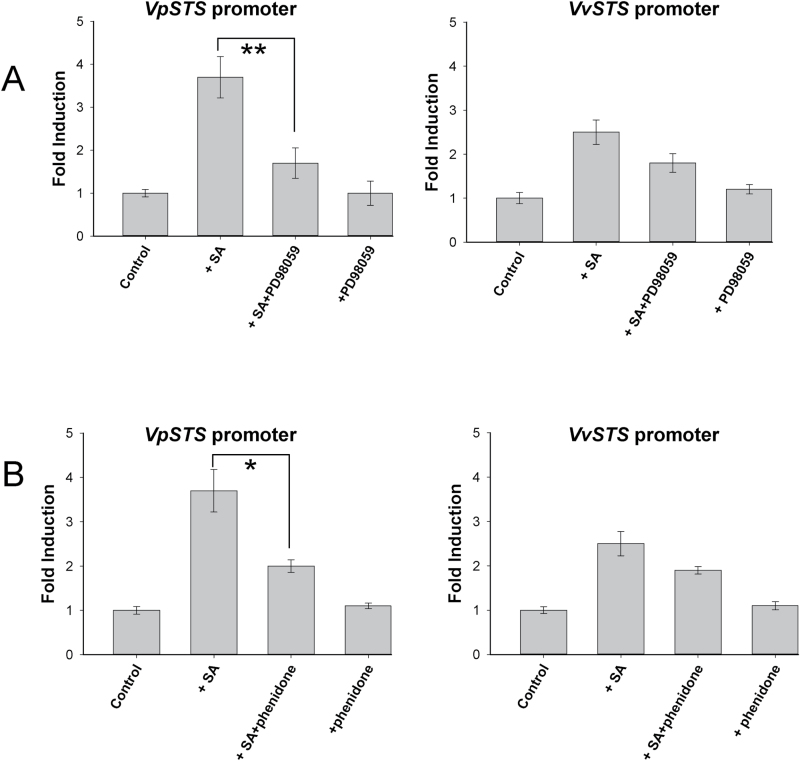
Activation of *STS* promoters by salicylic acid (SA) is modulated by MAPK and jasmonate signalling. Activation of *VpSTS* and *VvSTS* promoters by 50 µM SA was measured after pre-treatment with (A) the specific MAPK cascade inhibitor PD98059 (100 µM, added 30min prior to SA) or (B) the lipoxygenase inhibitor phenidone (2mM, added 30min prior to SA), which eliminates jasmonate acid synthesis, was used to block activation of *STS* promoters by SA. Data represent mean values and SEs from three biological replicates. * and ** indicate statistical signiﬁcance of the difference assessed by a one-sided paired *t*-test with confidence levels of *P*<0.05, or *P*<0.01, respectively.

The MAPK signalling cascade has been reported to act both upstream and downstream of the SA signalling pathway (Zhang and Liu, 2001; Zhang *et al.*, 2007), and was found to be necessary for induction of *STS* transcripts in grapevine (Chang and Nick, 2012). To explore whether the activation of *STS* promoters by SA is dependent on the MAPK pathway, the specific inhibitor PD98059 was used to suppress MAPK signalling in the SA treatment. When the transgenic suspension cells were pre-treated with PD98059 for 30min before the SA treatment, we found that the SA induction of both the *VpSTS* and *VvSTS* promoters was clearly, but partially, suppressed by the inhibitor of MAPK pathway (Fig. 9A). Compared with the induction by SA alone, the inhibitor reduced the activation of the *VpSTS* promoter from 3.7-fold to 1.7-fold, while the activation of the *VvSTS* promoter decreased from 2.5-fold to 1.8-fold (which was not significant). In the absence of SA, the MAPK inhibitor did not affect the promoter activities (Fig. 9A). Similarly to DPI, the inhibition by PD98059 remained partial, indicating that the MAPK cascade acts as a positive modulator of SA signalling to the *STS* promoters.

Since we had found that MeJA can induce the two *STS* promoter alleles to a similar extent (Fig. 7B), we probed for the contribution of JA to the promoter induction by SA, using phenidone, an inhibitor of lipoxygenases, because the inhibition of lipoxygenases quells the entire oxylipin metabolism including the synthesis of JA. In fact, we observed that the activation of both *STS* promoters by SA was affected after pre-treatment with 2mM phenidone for 30min (Fig. 9B): *VpSTS* promoter activation was decreased significantly from 3.7-fold to 2.0-fold, and *VvSTS* promoter activation from 2.5-fold to 1.9-fold, which is not significant. This finding indicates that synthesis of JA is a positive modulator of SA signalling to the *STS* promoters. As already found for DPI (Fig. 8B), the differential activation of the two promoter alleles by SA is not completely eliminated when MAPK activation (Fig. 9A) or JA synthesis (Fig. 9B) is inhibited. This means that these factors promote the SA effect by a second independent pathway.

## Discussion

Wild relatives of crop plants are often valuable genetic resources for breeding, because they have preserved resilience factors that have been lost during domestication due to biased selection for fast growth and high yield. For grapevine, resistance factors against biotrophic pathogens such as downy or powdery mildew have been successfully introgressed from wild American species (Eibach *et al.*, 2007; Gessler *et al.*, 2011). These resistance factors are generally interpreted in the context of ETI. Since both downy and powdery mildew of grapevine were introduced into Europe only in the 19th century and from there to Central and East Asia even later, ETI is not expected either in Non-American wild grapevine species or in *vinifera*. Nevertheless, several resistance factors have been identified in those grapevines that lack a co-evolutionary history with these pathogens: for downy mildew, the factor *Rpv10* from *V. amurensis* is already exploited in commercial *vinifera* varieties in Germany (Schwander *et al.*, 2012). As pointed out in detail in the Introduction, also for powdery mildew, several valuable resistance factors have been discovered in different wild Chinese grapevines as well as in the Uzbek *vinifera* variety ‘Kishmish Vatkana’. These factors also indicate that ETI may not be the only mechanism that can be exploited for resistance breeding.

The Chinese wild *V. pseudoreticulata* accession Baihe-35-1 was identified as a genotype with high resistance to powdery mildew (Wang *et al.*, 1995). Similar to the European Wild grapevine, *V. sylvestris*, an ETI-like mechanism is not very likely, since powdery mildew of grapevine is not native to China, but was introduced from either Europe or North America, with the first incidences in China reported in the 1950s (Wang, 1993; Wang *et al.*, 1995). However, since the accumulation of stilbenes resulting from induction of *STS* genes represents a crucial factor in the resistance to powdery mildew (Schnee *et al.*, 2008), we therefore addressed the role of a powdery mildew-responsive *STS* allele from the resistant Chinese wild *Vitis* species and used the susceptible *vinifera* cultivar ‘Carigane’ as reference. Grapevine *STS* genes are organized in a large family and can be activated by various abiotic and biotic factors (Parage *et al.*, 2012). Some members have been reported to respond to pathogen attack (Xu *et al.*, 2011; Dai *et al.*, 2012). The differential responsiveness of the two *STS* alleles is correlated with distinct differences in their promoter sequences, whereas the transcripts are fairly similar. Using transformation of *STS* promoter constructs driving a GUS reporter in Arabidopsis as the heterologous model, we can show that the specific differences in the *VpSTS* promoter are necessary and sufficient to confer high responsiveness to a compatible strain of powdery mildew, *G. cichoracearum* UCSC1, and that this elevated responsiveness is correlated with an elevated responsiveness to exogenous SA, whereas the response to MeJA is not altered. Expression of the full-length *STS* alleles in the same system shows that they confer accumulation of stilbenes in Arabidopsis accompanied by a partial resistance against the compatible strain of powdery mildew. Arabidopsis lacks the molecular and cellular machinery to accumulate stilbenes, which may be the reason why the stilbenes formed by the transformants are glycosylated. It remains to be elucidated to what extent they are cleaved in response to pathogen attack.

### A function for SA in basal immunity? Lessons from STS regulation

To understand the signalling upstream of *STS* and to integrate the data into the type of defence response (PTI versus ETI-like cell death-related immunity), we introduced the two promoter alleles into a promoter–reporter system (Höll *et al.*, 2013) based on a grapevine cell line derived from the *vinifera* cultivar ‘Pinot Noir’. In this cell line, the bacterial PAMP flg22 activates PTI, whereas the bacterial elicitor Harpin activates a cell death-related ETI-like response (Chang and Nick, 2012).

We found that flg22, but not Harpin, was able to activate *VpSTS* in this system, whereas *VvSTS* was not responsive. This places *VpSTS* in the context of basal immunity, consistent with the responsiveness of this promoter to MeJA (albeit that both alleles were responsive to the same degree). This finding is consistent with the observation that in the cell line used as host for the *VpSTS* promoter, flg22 causes the accumulation of JA and its bioactive conjugate JA-Ile, whereas Harpin fails to do so (Chang *et al.*, 2016). These observations are consistent with a model where *STS* is activated by basal immunity (possibly via activation of jasmonate signalling), rather than by cell death-related, ETI-like immunity.

On the other hand, the *VpSTS* promoter was significantly responsive to SA, and this responsiveness was much more pronounced compared with the *VvSTS* promoter, a pattern that had already been observed upon heterologous expression in Arabidopsis. Activation of the SA pathway is traditionally discussed in the context of cell death-related defence (Tenhaken and Rübel, 1997). Our results showed that induction of the *VpSTS* promoter by SA required calcium influx, NADPH oxidase activity, the MAPK pathway, and jasmonate synthesis, namely events that are typical for basal immunity.

The molecular events underlying early signalling seem to be mostly shared between PTI and ETI, including calcium influx, oxidative burst, or activation of MAPK cascades. On the other hand, flg22 and Harpin, while both activating *STS*, can generate a qualitatively different defence response in the grapevine cell line used for the promoter–reporter assay (Chang and Nick, 2012), leading to the question of at what point the bifurcation of basal and cell death-related immunity is generated.

Interestingly, we find that SA specifically activates the *VpSTS* promoter, as does flg22. Moreover, SA uses the same signalling events (calcium influx, NADPH oxidase-generated ROS, MAPK signalling, and jasmonate) that have already been found to activate flg22-induced accumulation of *STS* transcripts (Chang and Nick, 2012) as well as activation of *MYB14*, a transcription factor gene activating *STS* (Duan *et al.*, 2016). Addition of exogenous SA to tobacco suspension cells can induce a transient calcium influx (Kawano *et al.*, 1998), and MAPK signalling is activated by SA in tobacco (Zhang and Liu, 2001), whereas the link between SA and apoplastic oxidative burst seems not to be that straightforward (see, for example, Pogány *et al.*, 2009). Here, we found that both calcium and ROS signalling act downstream of SA (Fig. 8), because exogenous SA would over-ride the effect of Gd^3+^ and of DPI if SA were acting downstream of calcium and ROS signalling. Using a time course experiment, where the NADPH oxidase inhibitor DPI is administered at different time points with respect to the inducing SA (Supplementary Fig. S2), we see that the apoplastic oxidative burst is needed up to 60min after induction by SA (i.e. relatively late). It should be mentioned here that also for flg22-triggered basal defence, the apoplastic burst occurs later than calcium influx, whereas for Harpin-triggered cell death-related defence this sequence is reversed (Chang and Nick, 2012). These coincidences indicate a pattern whereby SA acts in the context of basal immunity. This conclusion is further supported by the fact that activation of the *VpSTS* promoter by SA can be blocked by the inhibitor PD98059 (Fig. 9A), which can block phosphorylation and activation of MAPKs (English and Cobb, 2002), as well as by phenidone, an inhibitor of JA synthesis (Fig. 9B).

We therefore arrive at a model where SA can activate the *STS* promoter through the same signalling machinery that is also activated by the PAMP flg22. The promoter allele of the wild Chinese grapevine can recruit this signalling more efficiently as compared with the allele found in the *vinifera* cultivar ‘Carigane’, and this efficient recruitment underlies the more efficient activation of stilbene accumulation in response to powdery mildew, which may be the reason why the investigated accession of the wild Chinese grapevine *V. pseudoreticulata* is resilient against powdery mildew. The reason for this superior recruitment of signalling for STS induction remains to be elucidated; however, a scenario where a member of the phenylpropanoid pathway (cinnamyl alcohol dehydrogenase) is modulated by an NB-LRR protein to culminate in powdery mildew resistance is also suggested by the analysis of the Ren1 factor from the Central Asiatic seedless variety ‘Kishmish Vatkana’ (Coleman *et al.*, 2009).

### Outlook: convergence of JA and SA signalling on the *STS* promoter

It has been shown that two R2R3-MYB-type transcription factor genes, *MYB14* and *MYB15*, can regulate the expression of STSs in grapevine (Höll *et al.*, 2013). Further analysis of a *MYB14* allele from *V. sylvestris* that was found to be correlated with high stilbene inducibility revealed that this promoter was activated by MeJA (as well as by UV light and flg22-triggered basal immunity), whereas SA was not effective (Duan *et al.*, 2016). Thus, there exist two pathways culminating in activation of the *STS* promoter: one pathway acts through activation of *MYB14* (presumably in concert with *MYB15*) in the context of basal immunity and is mostly independent of SA. The second pathway activates the *STS* promoter in a manner strongly dependent on SA (but also requires the same events transducing basal immunity as the *MYB14/MYB15*-dependent pathway). Therefore, we conjecture that additional transcription factors different from MYB14/MYB15 possibly participate in the regulation of *STS*. Promising candidates are the WRKY transcription factors that also act as regulators of SA-dependent defence responses (Wang *et al.*, 2006), and often are induced by SA. For instance, expression of *VvWRKY1* is regulated by SA in grapevine (Marchive *et al.*, 2007). Some *WRKY* transcription factor genes, such as rice *WRKY13* (Qiu *et al.*, 2007) or Arabidopsis *WRKY70* (Li *et al.*, 2004), can stimulate SA signalling to the cost of JA signalling. Recently, co-expression of specific *STS* members whose promoters contain a large number of W-box motifs with specific *WRKY* transcription factor genes has been reported for a drought-tolerant genotype of *vinifera* (Corso *et al.*, 2015). Since both *STS* promoters addressed in our study are rich in predicted W-box motifs (Fig. 1B) that are thought to act as binding domains for WRKY proteins, we will address the role of *WRKY* for the SA-dependent induction of *VpSTS*.

The classical dichotomy of a cell death-free, basal immunity (PTI), and a cell death-related, evolutionarily advanced immunity (ETI) has been questioned by transitional situations, suggesting that there is far more flexibility available than thought previously (Thomma *et al.*, 2011). Our finding that SA, which classically has been associated with cell death-related defence, can be recruited for basal immunity is consistent with this ‘blurred dichotomy’. We wonder whether the signalling function of SA as a molecule depends on the state to which (JA-dependent) basal immunity has progressed when SA is formed, an idea which we can address by modulating relative temporal patterns of early signals.

Independently of the molecular mechanism that will emerge from such studies, the current work shows that resistance against biotrophic pathogens such as powdery mildew can be achieved by multiple strategies: introgression of ETI-like HR-linked defence from North American wild grapes is certainly one of these strategies (Feechan *et al.*, 2013*a*), but boosting basal immunity to culminate in a swift and strong stilbene response might be an alternative strategy worth pursuing.

## Supplementary data

Supplementary data are available at *JXB* online.

Figure S1. Time course for the accumulation of the GUS reporter upon heterologous expression of *VpSTS::GUS* compared with *VvSTS::GUS* in transgenic Arabidopsis leaf tissues in response to treatment with 1mM SA or MeJA.

Figure S2. Response of two stilbene synthase promoters to SA with the time course of DPI quelling the increase of ROS abundance.

Table S1. Primers used for gene cloning and qRT-PCR for this study.

Supplementary Data
