# Supplementary material for: A stilbene synthase allele from a Chinese wild grapevine confers resistance to powdery mildew by recruiting salicylic acid signalling for efficient defence
Source: J Exp Bot. 2016 Oct 11;67(19):5841–56. doi: 10.1093/jxb/erw351 (PMC5066501; doi:10.1093/jxb/erw351)
Supplement: Supplementary Data [file supp_67_19_5841__index.html]

A stilbene synthase allele from a Chinese wild grapevine confers resistance to powdery mildew by recruiting salicylic acid signalling for efficient defence — A stilbene synthase allele from a Chinese wild grapevine confers resistance to powdery mildew by recruiting salicylic acid signalling for efficient defence — Supplementary Data 

# A stilbene synthase allele from a Chinese wild grapevine confers resistance to powdery mildew by recruiting salicylic acid signalling for efficient defence

## Supplementary Data

Data files

- supplementary\_figures\_S1\_S2.pdf - Supplementary Data
- supplementary\_table\_S1.pdf - Supplementary Data
