# Supplementary material for: A stilbene synthase allele from a Chinese wild grapevine confers resistance to powdery mildew by recruiting salicylic acid signalling for efficient defence
Source: J Exp Bot. 2016 Oct 11;67(19):5841–56. doi: 10.1093/jxb/erw351 (PMC5066501; doi:10.1093/jxb/erw351)
Supplement: Supplementary Data [file supp_erw351_supplementary_figures_S1_S2.pdf]

Supplementary data

A stilbene synthase allele from a Chinese wild grapevine confers resistance to Powdery Mildew by recruiting salicylic acid signalling for efficient defence

Yuntong Jiao[1,2,3,] , Weirong Xu[1,2,3], Dong Duan[4], Yuejin Wang[1,2,3], and Peter Nick[4*]

[1]College of Horticulture, Northwest A & F University, Yangling 712100, Shaanxi, People's Republic of China

[2]Key Laboratory of Horticultural Plant Biology and Germplasm Innovation in Northwest China, Ministry of Agriculture, Yangling 712100, Shaanxi, People's Republic of China

[3]State Key Laboratory of Crop Stress Biology in Arid Areas, Northwest A&F University, Yangling, Shaanxi 712100, People's Republic of China

[4]Molecular Cell Biology, Botanical Institute 1, Karlsruhe Institute of Technology, Kaiserstr. 2, D-78133 Karlsruhe, Germany

Figure S1

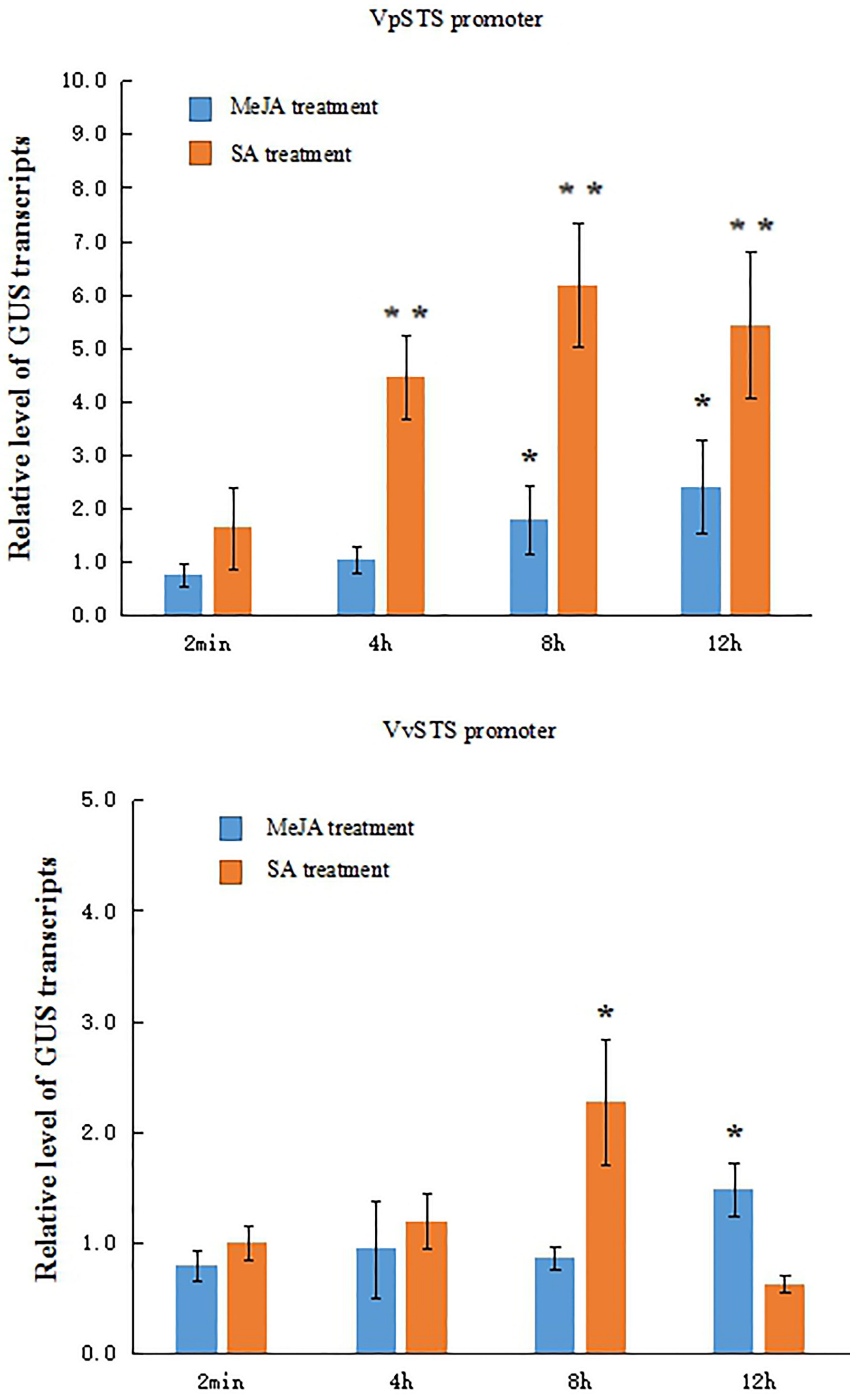

Figure S1. Time course for the accumulation of the GUS reporter upon heterologous expression of *VpSTS::GUS* (upper graph) compared to *VvSTS::GUS* (lower graph) in transgenic *Arabidopsis* leaf tissues in response to treatment with 1 mM of SA or MeJA. The transcript levels of *GUS* were normalized to *GAPDH* as an internal reference. All data are mean values from triplicate assays, standard errors is indicated on each bar. * and ** indicate statistically significance of the difference to the first time point by a one-sided paired t-test with confidence levels of $P<0.05$, or $P<0.01$, respectively.

Figure S2

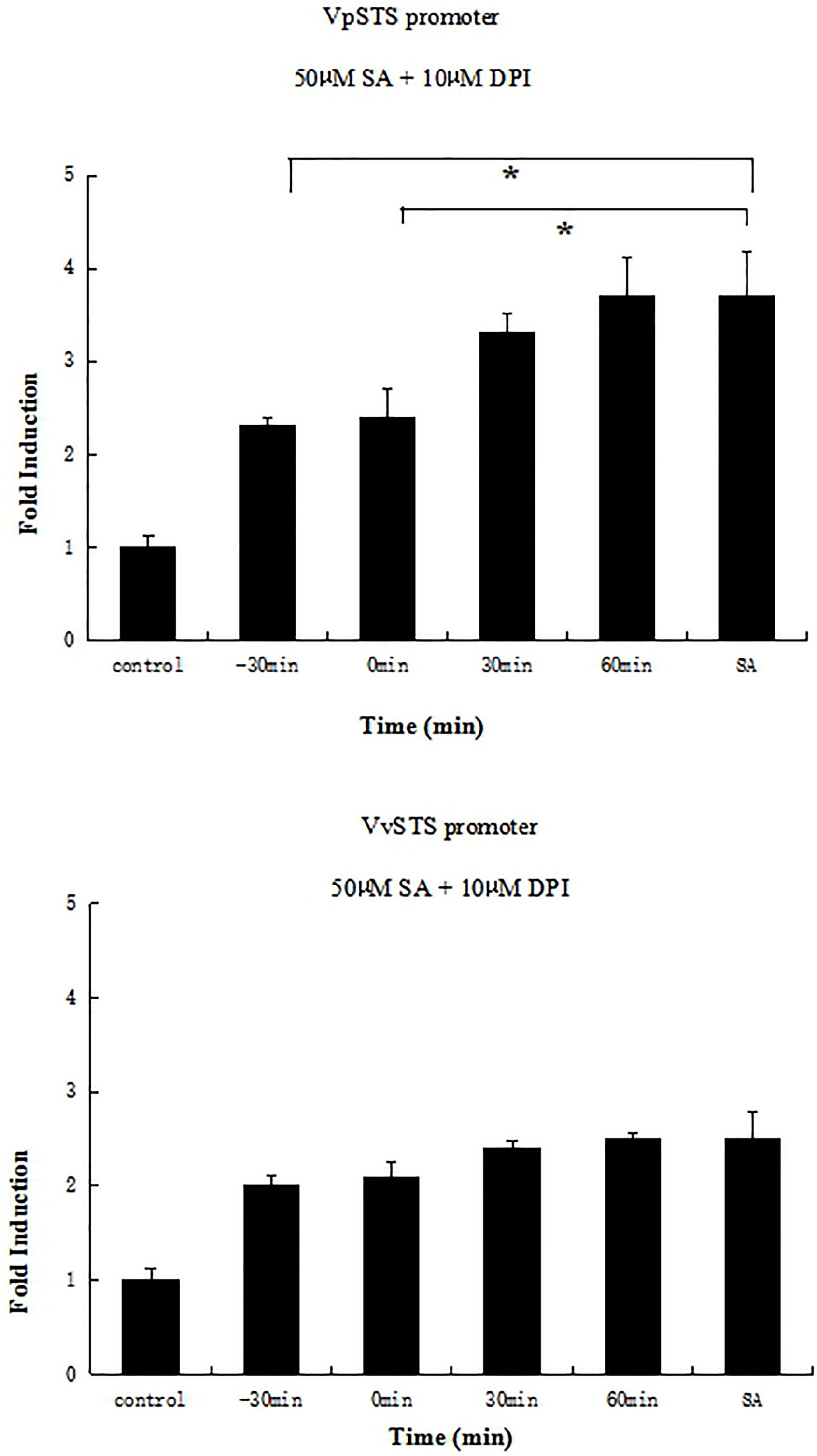

Figure S2. Response of two stilbene synthase promoters to SA with time course of DPI quelling the increase of ROS abundance. Column sequentially showing the solvent control; pretreatment with 10 μM DPI added 30 min before treatment with 50 μM SA; treatment with SA and DPI at the same time; adding DPI after treatment with SA for 30 min; and treatment with DPI after treatment with SA for 60 min. Data are mean values from three independent experiments and error bars show the standard errors, * indicates statistically significance of the difference assessed by a one-sided paired t-test with confidence level of $P<0.05$.
