# Supplementary material for: A stilbene synthase allele from a Chinese wild grapevine confers resistance to powdery mildew by recruiting salicylic acid signalling for efficient defence
Source: J Exp Bot. 2016 Oct 11;67(19):5841–56. doi: 10.1093/jxb/erw351 (PMC5066501; doi:10.1093/jxb/erw351)
Supplement: Supplementary Data [file supp_erw351_supplementary_table_S1.pdf]

Supplementary data

A stilbene synthase allele from a Chinese wild grapevine confers resistance to Powdery Mildew by recruiting salicylic acid signalling for efficient defence

Yuntong Jiao[1,2,3,] , Weirong Xu[1,2,3], Dong Duan[4], Yuejin Wang[1,2,3], and Peter Nick[4*]

[1]College of Horticulture, Northwest A & F University, Yangling 712100, Shaanxi, People's Republic of China

[2]Key Laboratory of Horticultural Plant Biology and Germplasm Innovation in Northwest China, Ministry of Agriculture, Yangling 712100, Shaanxi, People's Republic of China

[3]State Key Laboratory of Crop Stress Biology in Arid Areas, Northwest A&F University, Yangling, Shaanxi 712100, People's Republic of China

[4]Molecular Cell Biology, Botanical Institute 1, Karlsruhe Institute of Technology, Kaiserstr. 2, D-78133 Karlsruhe, Germany

Table S1.Primers used for this study

Primer Name	Primer sequence 5'-3'	Purpose
STS-F	5'-CGAGCTCGCTAATGATTCCAAATTCTAAATTTGAGATG-3'	The two fragments encoding the *STS* transcript and the respective native upstream promoter were amplified using these primers.
STS-R	5'-CCGCTCGAGTTAATTTGTAACCATAGGAACGCTATGCAGCA-3'	
Promoter-STS-F	5'-CGAGCTCGCTAATGATTCCAAATTCTAAATTTGAGATG-3'	To isolate the STS promoters.
Promoter-VpSTS-R	5'-CATGCCATGGGGATGCTAGATACGTACTGAAATTG-3'	
Promoter-VvSTS-R	5'-CATGCCATGGGGATGCTAGATACGTAATGAAATTG-3'	
attB-promoter-STS-F	5'-GGGGACAAGTTTGTACAAAAAAGCAGGCTTCGCTAATGATTCCAAATTCTAAATTT-3'	For GATEWAY cloning.
attB-promoter-VpSTS-R	5'-GGGGACCACTTTGTACAAGAAAGCTGGGTCGGATGCTAGATACGTACTGAAATTG-3'	
attB-promoter-VvSTS-R	5'-GGGGACCACTTTGTACAAGAAAGCTGGGTCGGATGCTAGATACGTAATGAAATTG-3'	
q-RT-PCR-GUS-F	5'-GAATACGGCGTGGATACGTTAG-3'	q-RT-PCR
q-RT-PCR-GUS-R	5'-GATCAAAGACGCGGTGATACA-3'	
q-RT-PCR-STS-F	5'-TGGGTCTTCAGCTGTGATTG -3'	
q-RT-PCR-STS-R	5'-GGCTCCTGCTGAATTAGGAATA-3'	
AtGAPDH-F	5'-TTGGTGACAACAGGTCAAGCA-3'	
AtGAPDH-R	5'-AAACTTGTCGCTCAATGCAATC-3'	
